# Negative Life Events and Problematic Internet Use as Factors Associated With Psychotic-Like Experiences in Adolescents

**DOI:** 10.3389/fpsyt.2019.00369

**Published:** 2019-05-29

**Authors:** Ju-Yeon Lee, Dahye Ban, Seon-Young Kim, Jae-Min Kim, Il-Seon Shin, Jin-Sang Yoon, Sung-Wan Kim

**Affiliations:** ^1^Chonnam National University Medical School, Gwangju, South Korea; ^2^Gwangju Bukgu Community Mental Health Center, Gwangju, South Korea

**Keywords:** psychotic-like experience, internet use, stress, coping, anxiety, depression

## Abstract

**Objectives:** Psychotic-like experiences (PLEs) and problematic internet use (PIU) are common in adolescents. However, little is known about the association between PLEs and PIU among adolescents. The present study examined the associations between PLEs and PIU and negative life events among adolescents.

**Methods:** In total, 1,678 adolescents attending high school were recruited for a cross-sectional survey. They completed self-reported assessments of PLEs using the Prodromal Questionnaire-16 (PQ-16) and measures of depression, anxiety, self-esteem, internet use, and negative life events using the Center for Epidemiological Studies Depression Scale (CES-D), the State-Trait Anxiety Inventory (STAI), the Rosenberg Self-Esteem Scale (RSES), the Korean Scale for Internet Addiction (K-scale), and the Lifetime Incidence of Traumatic Events for Children (LITE-C), including cybersexual harassment and school violence.

**Results:** A total of 1,239 subjects (73.8%) scored at least 1 on the PQ-16. The mean total and distress PQ-16 scores were significantly higher in students who used mental health services. The total and distress prodromal questionnaire-16 (PQ-16) scores were positively correlated with the CES-D, STAI-S, STAI-T, LITE-C, and K-scale scores but negatively correlated with the RSES score. Hierarchical linear regression analysis revealed that PLEs were significantly associated with a high K-scale score and the incidence of negative life events, such as LITE-C, cybersexual harassment, and bully–victims.

**Conclusion:** Our results demonstrate that PIU and negative life experiences were significantly associated with PLEs in adolescents. Assessment and therapeutic intervention with regard to internet use as a coping strategy for stress are needed to prevent the development of clinical psychotic symptoms.

## Introduction

Psychotic-like experiences (PLEs) are subclinical hallucinations and delusions that are common among adolescents (1) and are a manifestation of at-risk mental states (ARMS) for psychosis (2, 3). The presence of PLEs does not necessarily predict future conversion to psychosis (4), but it is important to address such experiences as they cause distress and functional impairment similar to individuals who transition to psychosis (5). Several psychosocial factors, such as depression, anxiety, poor self-esteem, and negative life experiences, have been reported to be risks for PLEs (6–9). In the context of negative life experiences, PLEs are associated with childhood trauma and recent life events (10, 11). Because adolescence is a distinct life stage with specific developmental tasks, adolescents must cope with various psychological events and bodily changes (12). In particular, school bullying creates substantial stress, especially during adolescence, when peer relationships become important. Cyberbullying (including sexual harassment) has become common, especially among teenagers who use the internet extensively (13). Research has revealed several serious mental health problems caused by the cyberbullying of adolescents (14).

The literature indicates that coping with the stress associated with PLEs may be important in terms of determining psychiatric outcomes (15, 16). Maladaptive coping is associated with the strong relationship between psychopathology and poor functioning in those with psychotic disorders (17, 18). Recent studies in adolescents have indicated that individuals who report subclinical psychotic experiences also commonly use poor coping styles (19). Addictive behaviors, including internet addiction, are known to be negative coping strategies with stressful events and deteriorate the individual functioning (20–22). Problematic internet use (PIU), conceptualized as “internet addiction,” is characterized by persistent compulsive use of the internet that interferes with daily life, leading to significant clinical impairment (23).

PIU is a serious public mental health problem worldwide, especially in adolescents (24). In addition, adolescents who consult for PIU have comorbid mental health problems (25). From a clinical perspective, it is important to explore mental health problems in adolescents with PIU and vice versa. A recent study showed that nonclinical PLE is positively associated with PIU in young adults (26). In our previous study, PIU in patients with a schizophrenia spectrum disorder was significantly associated with dysfunctional coping with stressful events. However, few studies have investigated the association between PLEs and PIU among adolescents. In particular, as South Korea has high-speed internet and a high rate of excessive smartphone use among teenagers (95.9%) (27), PIU is becoming a serious social problem, particularly among adolescents.

This study investigated the associations among PLEs, negative life events, and PIU in Korean community high school students. In particular, we assessed negative life events among adolescents due to not only childhood trauma but also recent stressful events including cybersexual harassment and bullying at school. This study will further facilitate our understanding of these issues in the community setting, including intervention and prevention for adolescents with PLE symptoms.

## Methods

### Study Design and Participants

This study was a community-based cross-sectional survey undertaken between July and September 2016 in Gwangju, Korea. In total, 2,013 first- and second-grade students from five high schools participated in the survey. Of the 2,013 students, 1,678 (83.4%) were included in the analyses after excluding incomplete or inappropriate responses on the scales. This survey was approved by the principal of each school, and the sample comprised students who voluntarily agreed to complete the questionnaires with informed consent. All measures, including data on sociodemographic characteristics, were self-administered. The Institutional Review Board of Chonnam National University Hospital approved the study. All participants gave written informed consent prior to participation in the study.

### Measures

*Sociodemographic characteristics*. Gender, age, religion, and academic achievement information were obtained from the students. The students were asked about their experience using mental health services offered by a psychiatric clinic, a community center, a counseling center, and a Wee Center (the name given by the Education Office of Korea); the Wee Center offers programs and counseling to city-based students with mental health problems.

*Psychotic-like experiences*. The Prodromal Questionnaire-16 (PQ-16) is a brief self-report screening questionnaire that assesses the presence of positive and negative symptoms on a two-point scale (true/false) (28). The total score on the PQ-16 was calculated by adding up the agreed items. For each endorsed item, distress was rated on a four-point scale (ranging from no distress to high distress). The distress scale ranged from 0 to 96. The validity of the Korean version of the PQ-16 has been well established (29).

*Depression and anxiety*. Depression was measured using the Center for Epidemiological Studies Depression Scale (CES-D) (30). The CES-D contains 20 items regarding depressive symptoms experienced in the past week that are rated on a Likert-type scale [“less than 1 day” to “most or all (5–7) days”]. Possible total scores range from 0 to 60, and a higher score reflects greater depression. The reliability and validity of the Korean version of the CES-D have been well established (31). Anxiety was measured using the State-Trait Anxiety Inventory for Children (STAIC) (32). The STAIC consists of two independent domains with 20 items that measure state (STAI-S) and trait (STAI-T) anxiety levels on a three-point Likert scale. Total scores range from 20 to 60 on each domain, with higher scores indicating a higher level of anxiety. The reliability and validity of the Korean version of the STAIC have been well established (33).

*Self-esteem*. To measure self-esteem, we used the Rosenberg’s Self-Esteem Scale (RSES), which contains 10 items on a four-point Likert scale. A higher score indicates more positive self-esteem (34).

*Internet use*. PIU was assessed by the short-form Korean Scale for Internet Addiction (K-scale) for adolescents, which was developed by the Korea National Information Society Agency and has been validated in a Korean population (35). The scale consists of 15 items measuring daily life disturbances, virtual interpersonal relationships, deviant behaviors, withdrawal, and tolerance; items are rated on a four-point Likert-type scale.

*Negative life events*. Lifetime Incidence of Traumatic Events–Child (LITE-C) is a self-checklist that assesses loss and traumatic experiences in children and adolescents (36). The score is calculated by adding the number of “yes (presence)” responses to 16 types of trauma in the LITE-C. In addition to the LITE-C, we added some negative life events, such as cyber harassment and bullying; we included victims, witnesses, and bully–victims.

### Statistical Analyses

We calculated PQ-16 numbers and percentages. The mean score differences between various categorical sociodemographic factors were analyzed using the *t*-test and analysis of variance (ANOVA). Descriptive statistics were employed to estimate the means and standard deviations of continuous variables. Pearson’s correlation coefficient analysis was performed to evaluate the relationships between clinical variables and PQ-16 scores. We used hierarchical linear regression to examine the effects of clinical status, negative life events, and PIU, which were related to PQ-16 score in univariate analyses on PQ-16 score. In step 1, participants’ clinical information (depression, anxiety, and self-esteem), which were significantly correlated with dependents, were entered. In step 2, negative life experiences including LITE-C, which were significantly differences in PQ-16 score tested by *t*-test or Pearson’s correlation, were entered. Final step, K-scale indicating PIU that was significantly correlated with dependents, was entered. Output results including R^2^, R^2^-changes, F value, and standardization regression coefficient (ခβ) were provided in the regression models. Statistical Package for the Social Sciences (SPSS) for Windows software (ver. 21.0; SPSS Inc., Chicago, IL, USA) was used to perform the statistical tests. All statistical tests were two-tailed, and *p*-values <0.05 were considered significant.

## Results

**Figure 1:**
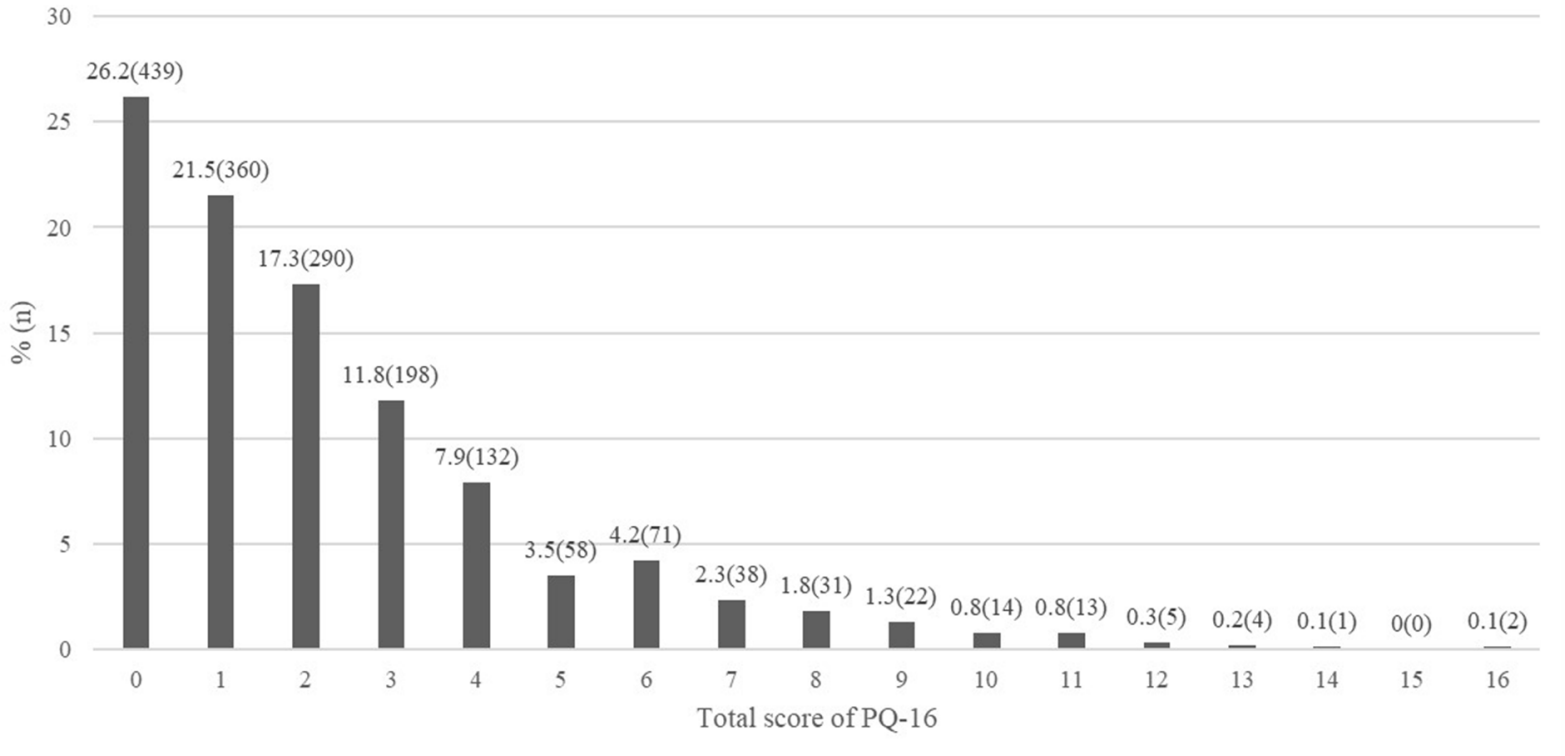
Percentage of number on the total score of PQ-16 (*n* = 1,678).

Of the 1,678 students, 1,078 were boys (64.2%) and 600 were girls. The mean age was 18.6 ± 0.5 years. The mean total and distress scores on the PQ-16 were 2.3 ± 2.6 and 38.0 ± 3.0, respectively. The distribution of PQ-16 total scores is shown in **Figure 1**. A total of 1,239 subjects (73.8%) scored at least 1 on the PQ-16; 11.9% scored 6 or more, indicating the clinical significance of ARMS. **Table 1** shows the mean differences in PQ-16 scores by demographic factors. We found no significant difference in PQ-16 scores by gender, religion, or academic achievement. The mean total and distress PQ-16 scores were significantly higher in students who used mental health services. The Wee Center was the most frequently visited institution (*n* = 87), followed by the community center (*n* = 46), the counseling center (*n* = 34), and the psychiatric clinic (*n* = 29). In terms of negative life events, students who experienced cybersexual harassment scored significantly higher in total and distress PQ-16 scores. Both mean scores were significantly higher for students who experienced school violence as victims, witnesses, and bully–victims.

**Table 1:** Sociodemographic factors that influence PQ-16 score.

		PQ-16 total score			PQ-16 distress score		
		Mean (SD)	Statistics	p	Mean (SD)	Statistics	p
Gender	N (%)						
Male	1,078 (64.2)	2.3 (2.5)	−0.367	0.714	3.0 (3.8)	−0.581	0.561
Female	600 (35.8)	2.4 (2.6)			3.1 (4.0)		
Religion							
Yes	655 (39.0)	2.5 (2.7)	1.640	0.101	3.2 (4.1)	1.571	0.116
No	1,023 (61.0)	2.3 (2.4)			2.9 (3.7)		
Academic achievement							
Good	590 (35.4)	2.5 (2.6)	4.518	0.104	3.2 (3.8)	5.445	0.066
Average	527 (31.4)	2.2 (2.5)			2.8 (3.8)		
Poor	550 (32.8)	2.3 (2.6)			2.9 (4.0)		
Use for mental health service							
(−)	1,523 (90.8)	2.2 (2.4)	−5.768	<0.001	2.8 (3.5)	−5.876	<0.001
(+)	155 (9.2)	3.5 (3.4)			4.7 (6.3)		
Negative life events							
Cybersexual harassment							
(−)	1,505 (89.7)	2.2 (2.4)	−7.201	<0.001	2.8 (3.7)	−6.044	<0.001
(+)	172 (10.3)	3.7 (3.0)			4.7 (4.7)		
Bullying, victims							
(−)	1,503 (89.6)	2.1 (2.3)	−10.327	<0.001	2.7 (3.4)	−11.114	<0.001
(+)	174 (10.4)	4.2 (3.4)			6.0 (5.9)		
Bullying, witness							
(−)	1,327 (79.2)	2.0 (2.2)	−12.129	<0.001	2.4 (3.1)	−12.117	<0.001
(+)	349 (20.8)	3.8 (3.1)			5.2 (5.4)		
Bullying, bully–victims							
(−)	1,577 (94.0)	2.2 (2.4)	−11.867	<0.001	2.7 (3.4)	−12.893	<0.001
(+)	101 (6.0)	5.1 (3.5)			7.6 (6.4)		

PQ-16, Prodromal Questionnaire-16.

Correlational analyses revealed that both the total and distress PG-16 scores were positively correlated with the CES-D, STAI-S, STAI-T, LITE-C, and K-scale scores and negatively with the RSES score (**Table 2**). The results of the hierarchical multiple regression analyses for PQ-16 score are shown in **Table 3**. The hierarchical multiple regression for PQ-16 total score revealed that at step 1, clinical information (depression, anxiety, and self-esteem) contributed significantly to the regression model, *F* (4,15) = 147.72, *p* < 0.001, and accounted for 28.1% of the variation in PQ-16 total score. Adding negative life events to the regression model explained an additional 8.5% of the variation in PQ-16 total score, and this change in R^2^ was significant, *F* (5,15) = 40.58, *p* < 0.001. Finally, the addition of K-scale to the regression model explained an additional 1.0% of the variation in PQ-16 total score, and this change in R^2^ was also significant, *F* (1,15) = 25.07, *p* < 0.001. The independent variables accounted for 37.7% of variance in PQ-16 total score. The hierarchical multiple regression for PQ-16 distress score revealed that at step 1, clinical information contributed significantly to the regression model, *F* (4,15) = 147.68, *p* < 0.001, and accounted for 28.0% of the variation in PQ-16 distress score. Adding negative life events to the regression model explained an additional 7.8% of the variation in PQ-16 distress score, and this change in R^2^ was significant, *F* (5,15) = 37.10, *p* < 0.001. Finally, the addition of K-scale to the regression model explained an additional 0.9% of the variation in PQ-16 distress score, and this change in R^2^ was also significant, *F* (1,15) = 22.68, *p* < 0.001. The independent variables accounted for 36.8% of variance in PQ-16 distress score. Ultimately, high scores on the CES-D, STAI-T, K-scale, and LITE-C scale; low scores on the RSES scale; and status as a bully–victim were strongly associated with both the total and distress PQ-16 scores. Cybersexual harassment predicted the total PQ-16 score but not the distress score.

**Table 2:** Clinical measures and their Pearson’s correlation coefficients with PQ-16 score.

Scales (mean ± SD)	PQ-16 total score	PQ-16 distress score
CES-D (12.2 ± 9.3)	0.435**	0.449**
STAI-S (39.1 ± 8.9)	0.184**	0.184**
STAI-T (31.2 ± 7.8)	0.514**	0.515**
RSES (26.9 ± 5.5)	−0.185**	−0.190**
LITE-C (2.6 ± 2.1)	0.404**	0.388**
K-scale (25.3 ± 7.1)	0.314**	0.309**

CES-D, Center for Epidemiological Studies Depression Scale; STAI, State-Trait Anxiety Inventory; RSES, Rosenberg Self-Esteem Scale; LITE-C, Lifetime Incidence of Traumatic Events-Child; K-scale, Korean Scale for Internet Addiction.

**p < 0.001.

**Table 3:** Hierarchical linear regression analyses predicting PQ-16 score.

	PQ-16 total score			PQ-16 distress score		
	Step 1(嫒β)	Step 2(嫒β)	Step 3(嫒β)	Step 1(嫒β)	Step 2(嫒β)	Step 3(嫒β)
Independent variables						
CES-D	0.173***	0.144***	0.140***	0.189***	0.159***	0.156***
STAI-S	0.011	0.021	0.019	−0.003	0.007	0.004
STAI-T	0.425***	0.355***	0.325***	0.413***	0.346***	0.317***
RSES	0.090**	0.073*	0.073*	0.083*	0.067*	0.068*
LITE-C		0.256***	0.245***		0.237***	0.227***
Cybersexual harassment		0.057*	0.053*		0.036	0.032
Bullying, victims		−0.041	−0.045		−0.056	−0.060
Bullying, witness		0.001	−0.006		−0.004	−0.010
Bullying, bully–victims		0.083*	0.091**		0.128***	0.136***
K-scale			0.110***			0.105***
R^2^	0.281	0.366	0.377	0.280	0.358	0.368
∆R^2^	0.281	0.085	0.010	0.280	0.078	0.009

*p < 0.05, **p < 0.01, ***p < 0.001.

## Discussion

The fact that a high percentage of participants scored at least 1 on a PQ-16 item (73.8%) is consistent with previous research in young community samples (37). Also, the percentage of students who scored at least 6 on positive PQ-16 items (indicative of clinical significance) was 11.9%, thus higher than the prevalence rate of 5–8% in the general population (38, 39). This result is in line with the prevalence of PLEs in adolescents (about 10%; thus generally higher than in adults) (40, 41).

In this study, students who used a mental health service had higher mean PQ-16 total and distress scores. It is possible that students with a variety of mental health problems, such as emotional and behavioral difficulties, use mental health services. PLEs are more frequent in help-seeking subjects (42, 43). In particular, PLE-associated distress may encourage help-seeking behavior (44). Our findings suggest that students with mental health problems and PLEs tend to have help-seeking behavior. Furthermore, individuals seeking help to deal with their PLEs could be viewed as being in the prodromal phase of various disorders. However, our results show that approximately half of students who used mental health services were referred to the Wee Center, which offers counseling services to students. Our recent study showed that school counselors are unfamiliar with the concept of high risk for psychosis and lack of confidence in treating those who have had PLEs (45). Early detection and timely delivery of psychiatric services for those who have PLEs is important to prevent a delay in the implementation of an early intervention. In this regard, teachers and school counselors should be provided with proper education regarding students with PLEs.

The severity levels of PLEs and distress as measured by the PQ-16 were associated with psychological difficulties, such as depression, anxiety, and low self-esteem. These results are in line with those of previous studies showing that PLEs were associated with various psychopathologies (46–49). Depression increased the risk of transition to a full-blown psychotic disorder in the ARMS study (50). One previous work suggested that the association between PLEs and poor functioning might be explained by the extent of depression (42). We found that low self-esteem was significantly associated with both total and distress PQ-16 scores. One cognitive model of psychosis suggests that a low self-concept is related to the development and maintenance of PLEs (51). A recent study indicated that those experiencing more hallucinatory-like events exhibited lower levels of self-esteem (52). In our study, trait anxiety was significantly associated with PLEs according to the regression analysis. Trait anxiety serves as a proxy for proneness in those who experience maladaptive anxiety (53); those who score high in “trait anxiety” exhibit differential processing of threatening information. This tendency is termed “attentional threat bias”; one previous study reported that trait anxiety significantly influenced the relationship between cognitive bias and PLEs (54). Our results suggest that biased cognitive processes in adolescents exhibiting high levels of trait anxiety may independently affect PLEs.

Although most PLEs experienced during adolescence are transitional in nature, 20% of subjects experience persistent PLEs, and 7% develop psychotic disorders in adulthood (55). Psychopathologies, such as depression, anxiety, and poor self-esteem, should be routinely assessed and considered for treatment given that they may contribute to later psychosis.

In this study, students who had negative life events, including losses or trauma and recent stressful events, such as cybersexual harassment and school violence, exhibited higher total and distress PQ-16 scores. A strong body of literature has addressed the role of childhood trauma as one of the risk factors for developing a psychotic illness in adolescence (46, 56). Furthermore, although many researchers investigating the relationship between trauma and psychosis have focused on the role of childhood adversity, there is growing evidence for a role of recent stressful life events in the development of psychosis (10, 57). Accordingly, in our study, cybersexual harassment and bullying were significantly associated with PLEs in adolescents. Cybersexual harassment is a type of internet abuse, which can take various forms, such as unsolicited posts and comments on social media sites. There is evidence that individuals with unwanted sexual experiences are at higher risk for developing PLEs (58). Public attention has focused on adolescent internet-mediated victimization, including unwanted exposure to online pornography and sexual messaging, which increase their vulnerability to sexual victimization (59). Our research suggests that cybersexual harassment may be a negative and threatening event that is predictive of PLEs in adolescents.

In particular, the regression analysis showed that the bully–victim status after school violence was a predictor of PLEs. Our findings support previous studies indicating that the prevalence of PLEs is greater in adolescents who have been exposed to school violence, including both victims and perpetrators of bullying (46). This finding suggests that traumatic experiences related to school violence seem to have a salient impact on PLEs, particularly during adolescence when peer relationships become critical to consolidate personal identity (60, 61).

In our study, the PLE was significantly associated with PIU measured by the K-scale in the regression analysis. PIU can be recognized as a maladaptive way of coping with life’s stressors (20, 21). Several studies have found that ARMS individuals tend to engage in more maladaptive coping than nonpsychiatric controls (62, 63). Furthermore, there is some evidence that individuals with schizophrenia experience a disruption in the biological system that responds to stress (64, 65). In a population of stabilized patients with schizophrenia, PIU was significantly associated with ineffective coping strategies to alleviate stress (66). Our findings suggest that the development of PLEs is associated with maladaptive coping strategies during the process of responding to stressful events. This is the first study to determine the relationship between PLEs and PIU among adolescents. Specialized interventions, including problem solving and coping skills training, are needed to help adolescents who have more access to the internet to exploit the internet as a positive coping strategy.

Our study had several limitations. First, this study was cross-sectional; therefore, longitudinal studies are required to confirm the directionality of the relationships in the present analysis, particularly between PIU and PLEs. Additionally, as it is probable that PLEs could make adolescents more likely to be victimized, we may have underestimated the associations between PLEs and negative life events. Second, the generalizability of the results is limited given the targeting of recruited students from one community. Third, we relied on self-reported measures and did not conduct clinical interviews, limiting the clinical validity of the data.

In conclusion, PLEs among adolescents were likely to co-occur with emotional problems, particularly depression, trait anxiety, and low self-esteem. In addition, this study highlights the associations among PLEs, negative life events, and PIU in community adolescents. Our results suggest that a number of traumatic events, including cybersexual harassment and bullying, may increase the risk of PLEs among adolescents. PIU, a maladaptive strategy used to cope with negative life events, was associated with PLEs in adolescents. These have potentially important clinical implications to manage and help adolescents with traumatic events and PIU to prevent the development of more serious clinical psychosis.

## Ethics Statement

This survey was approved by the principal of each school, and the sample comprised students who voluntarily agreed to complete the questionnaires with informed consent. All measures, including data on sociodemographic characteristics, were selfadministered.

The Institutional Review Board of Chonnam National University Hospital approved the study. All participants gave written informed consent prior to participation in the study.

## Author Contributions

SWK and JYL were involved in the conception and design of the study. DB conducted the data collection. SWK and JYL were involved in the analysis and drafted the manuscript. JMK, SYK, ILS, and JSY revised the manuscript critically for important intellectual content. All authors contributed to and have approved the final manuscript.

## Funding

This study was supported by a grant of the Korean Mental Health Technology R&D Project, Ministry of Health & Welfare, Republic of Korea (HM15C1140).

## Conflict of Interest Statement

The authors declare that the research was conducted in the absence of any commercial or financial relationships that could be construed as a potential conflict of interest.
